# A novel catalyst system for the synthesis of N,N′-Methylenebisacrylamide from acrylamide

**DOI:** 10.1080/15685551.2017.1332138

**Published:** 2017-05-26

**Authors:** Abdullah Avşar, Yener Gökbulut, Burak Ay, Selahattin Serin

**Affiliations:** ^a^ Arts and Science Faculty, Department of Chemistry, Çukurova University, Adana, Turkey

**Keywords:** N,N′-Methylenebisacrylamide, catalyst, monomer synthesis, Cu(II) complex

## Abstract

N,N′-Methylenebisacrylamide (MBAA), which is an important raw material in a widely industrial area, was synthesized using convenient catalysts. The synthesized monomer was fully characterized by the elemental analysis, fourier transform infrared (FT-IR) spectroscopy, thermogravimetric analysis (TGA), ^1^H-NMR, ^13^C-NMR, GC/MS, and high performance liquid chromatography (HPLC) analysis. Six different homogeneous and heterogeneous catalysts were used to obtain maximum monomer yield. The MBAA was obtained with 95% yield by using Cu(II) catalyst containing carboxylate groups ligands.

## 1. Introduction

**Scheme 1.**
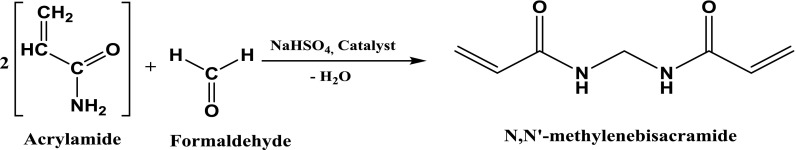
Structure of synthesized MBAA.

MBAA is a molecule that is used as a crosslinking agent in chemical reactions during the formation of polymers. Acrylamide is one of the most common compounds that use MBAA during the polymerization reactions. The bisacrylamide part of the molecule polymerizes with acrylamide and creates a crosslinked structure as opposed to a linear structure. This crosslinking imparts higher strength and toughness to the polymer in its end-use applications. Being a crosslinking agent, MBAA is used in various polymerization reactions to produce polymers and copolymers [1–10]. As a result, this molecule is in great demand in the chemical industry. The growing need for these polymers in waste water treatment, gel electrophoresis, papermaking, ore processing, tertiary oil recovery and the manufacture of permanent press fabrics has been one of the most important factors governing the demand for MBAA. The debated carcinogenic nature of acrylamides in food has been one of the factors restraining the market in this segment. However, the growing need for stable copolymers and composites is expected to move the market for MBAA along steadily in the near future. Although there are other molecules that can be used as substitute materials for MBAA, its versatility and stability in most polymerization reactions related to acrylonitrile boosts the potential for this molecule in the global market. MBAA can be obtained using acrylamide and formaldehyde in the presence of catalysts (Scheme 1). We report the synthesis of monomer with some types of Cu(II), Fe(II), Ni(II) and Pd(II) catalysts. We want to achieve the synthesis of MBAA which is an important raw material in a widely industrial area such as adhesives, coatings, thickeners etc. [11–13]. Methods for the synthesis of some of these compounds have been the tittle in some previous patents, but the yield of synthesis was not enough [14]. We tried several catalysts for the synthesis of the mentioned compound with different yields. In this study, we achieved synthesize of MBAA by using Cu(II) carboxylate catalyst with 95% yield, which is a high and remarkable percentage. No information has been given about the catalyst structure due to make a patent application.

## 2. Experimental

### 2.1. Materials and methods

All chemicals were purchased from a major chemical supplier as high or highest purity grade and used without further purification. Catalysts were synthesized in the laboratory. Melting points were determined with a Digital Melting Point Gallenkamp. IR spectra were measured with Thermo Scientific Nicolet IS10 Model FT-IR Spectrophotometer using ATR method with a resolution of 4 cm^−1^ at room temperature. HPLC analysis was performed for the characterization and purity of MBAA. For the HPLC analysis a Shimadzu HPLC system equipped with a reversed phase C8 column (250 cm × 4.6 mm column dimensions, 5 μm particle sizes, Ascentis®) was used. CH_3_OH:CH_3_CN:H_2_O (50:20:30, v/v) was used for the separation of the mobile phase. The column temperature was 35 °C, the detection wavelength was 254 nm, the flow rate was 1.0 mL min^−1^ and the retention time was 15 min. TGA analysis was conducted in nitrogen atmosphere with Perkin Elmer Pyris Diamond TG/DTA equipment at a heating rate of 10 °C min^−1^. ^1^H-NMR and ^13^C-NMR analysis were recorded wit a Bruker Avance III HD 600 MHz Ultra Shield TM. GC/MS analysis was performed by using Thermo Brand chromatograph with TR5MS capillary columns.

### 2.2. Synthesis of MBAA using Cu(II) acetate as a catalyst

A solution of acrylamide (19.90 g, 280 mmol), formaldehyde (4.20 g, 140 mmol), copper(II) acetate (0.10 g, 0.550 mmol), and concentrated HCl (2 mL, 65.65 mmol) were added succesively into the flask. The mixture was refluxed for 90 min and then cooled at room temperature. The solution mixture was separated from the solid phase and dried at room temperature. Yellow solid obtained was 11.20 g. Yield (52%), M. P. > 300 °C. It is interesting to note that when using NaHSO_4_ (1.60 g, 13.33 mmol) instead of HCl refluxed 2 h, the yield was increased and 12.78 g yellow solid was obatined. Yield was 60%, M. P. > 300 °C.

### 2.3. Synthesis of MBAA using Cu(II) glyoxime as a catalyst

A solution of acrylamide (19.9 g, 280 mmol), formaldehyde (4.20 g, 140 mmol), Cu(II) glyoxime (0.10 g, 0.66 mmol), and NaHSO_4_ (1.60 g, 13.33 mmol) were added succesively into the flask. The mixture was refluxed for 2 h and then cooled at room temperature. The solution mixture was separated from the solid phase and dried at room temperature. Yellow solid obtained was 11.20 g. Yield (52%), M. P. > 300 °C.

### 2.4. Synthesis of MBAA using Pd(II) glyoxime as a catalyst

The preparation of MBAA was similar to that of the synthesis 2.3 except that Pd(II) glyoxime (0.10 g, 0.51 mmol) was used instead of copper(II)-glyoxime.No conversion was observed.

### 2.5. Synthesis of MBAA using Fe(II) glyoxime as a catalyst

The preparation of MBAA was similar to that of the synthesis 2.3 except that Fe(II) glyoxime (0.10 g, 0.69 mmol) was used instead of copper(II) glyoxime. No conversion was observed.

### 2.6. Synthesis of MBAA using Ni(II) glyoxime as a catalyst

The preparation of MBAA was similar to that of the synthesis 2.3 except that Ni(II) glyoxime (0.10 g, 0.68 mmol) was used instead of copper(II) glyoxime. No conversion was observed.

### 2.7. Synthesis of MBAA using Cu(II) carboxylate as a catalyst

The preparation of MBAA was similar to that of the synthesis 2.3 except that Cu(II) carboxylate (0.10 g, 0.12 mmol) was used instead of copper(II) glyoxime. All ingredient were mixed as mentioned above. The mixture was refluxed (80–85 ^°^C) for about two hours then cooled, was filtered out and dried. Yellow solid obtained was 19.85 g. Yield (95%), M. P. > 300 °C. Anal. Calcd. for C_7_H_10_N_2_O_2_: C, 54.54; H, 6.54; N, 18.17. Found: C, 54.10; H, 6.14; N, 18.34%. IR data (cm^−1^): 3300(m), 3065(w), 2955(w), 1656(s), 1625(s), 1538(s), 1408(s), 1383(s), 1304(s), 1225(s), 1120(s), 989(s).

## 3. Results and discussion

### 3.1. IR spectra

**Figure 1.**
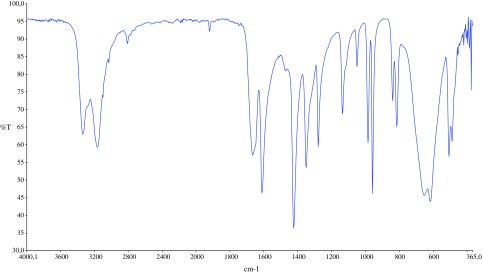
Infrared spectra of acrylamide.

**Figure 2.**
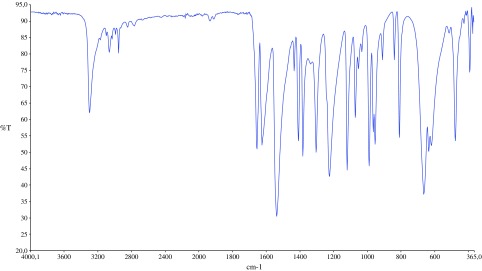
Infrared spectra of MBAA.

The infrared spectra of the acrylamide and target compound are shown in Figures 1 and 2. The characteristic two N–H stretch absorptions of the primary amine vibrations in the free acrylamide are found between 3300 and 3500 cm^−1^ (Figure 1). In the infrared spectra of MBAA (Figure 2), absorption band at 3302 cm^−1^ is assigned to the stretching vibrations of υ(N–H) in the molecule. The absorption related to the υ(C=CH) and υ(C–H) stretching vibrations of the MBAA were observed at 3065 and 2955 cm^−1^, respectively [15]. The presences of characteristic bands between 1360 and 1080 cm^−1^ are assigned to absorption vibrations of υ(C=O). The obtained spectral data of MBAA is in good agreement with the HPLC data of MBAA.

**Figure 3.**
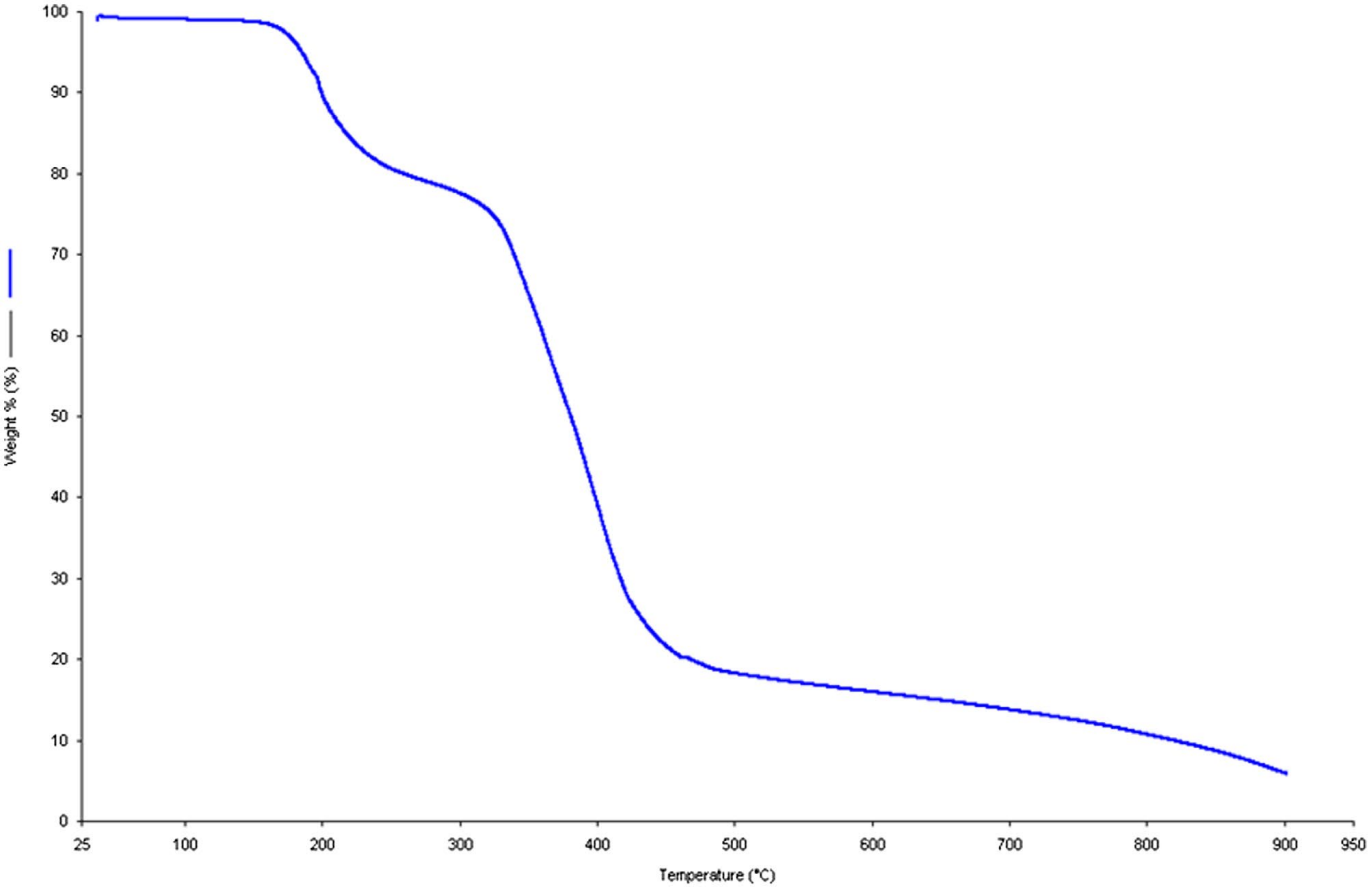
TGA curve of synthesized monomer.

**Figure 4.**
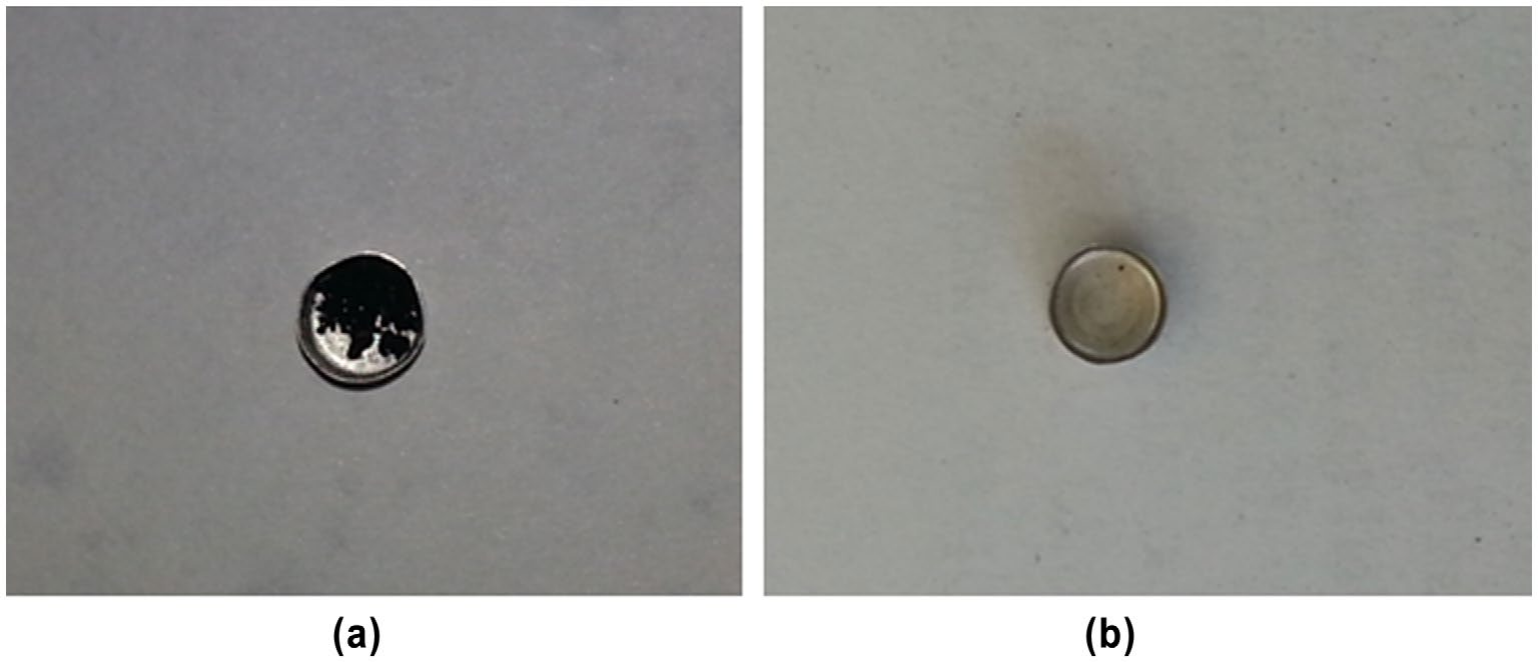
Carbonized MBAA at 900 °C under nitrogen atmosphere (a). Fully decomposed MBAA at 900 °C under oxygen atmosphere (b).

**Figure 5.**
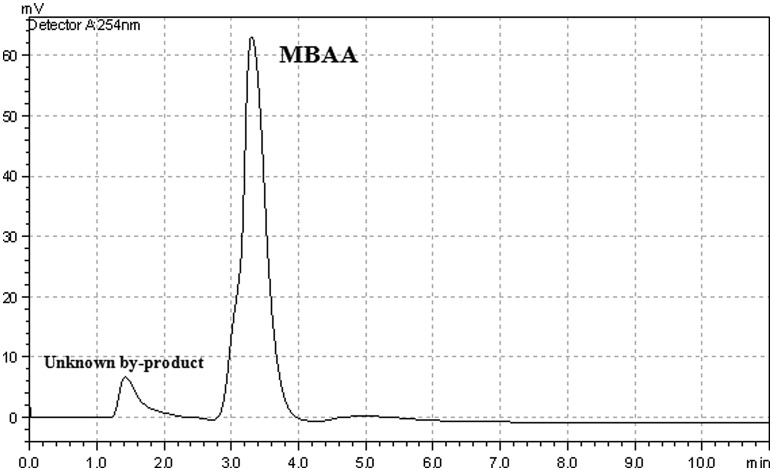
HPLC chromatogram of the reference sample of MBAA.

**Figure 6.**
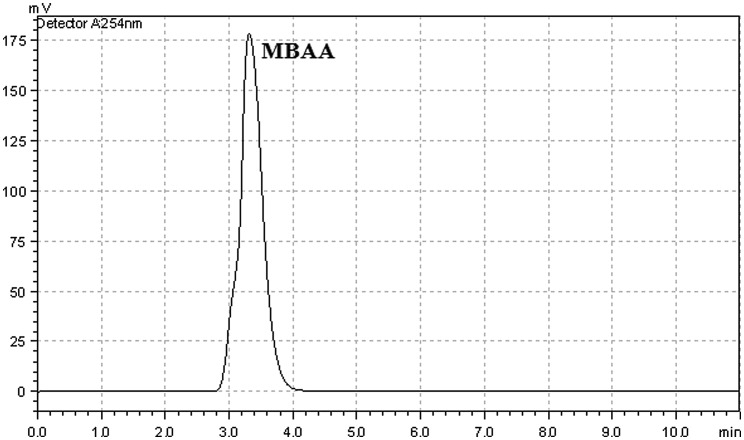
HPLC chromatogram of the of MBAA using Cu(II) catalyst.

**Figure 7.**
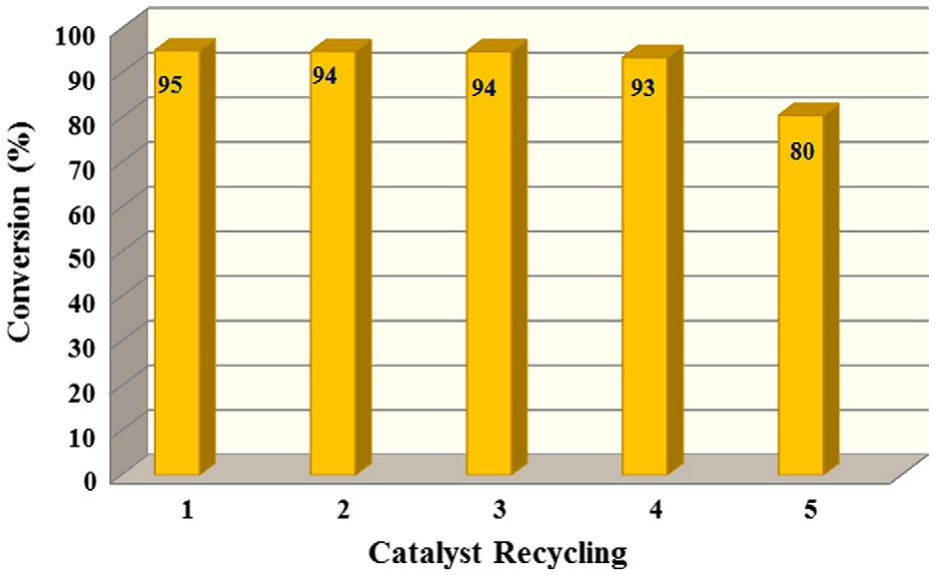
The reusability of the Cu(II) catalyst.

### 3.2. ^1^H-NMR and ^13^C-NMR analysis

**Scheme 2.**
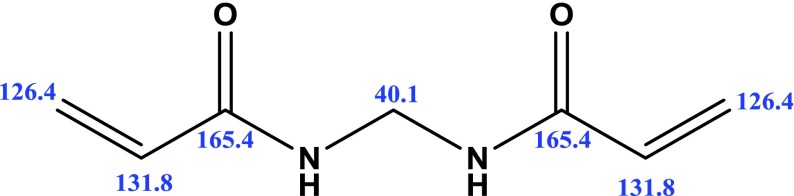
Experimental ^13^C-NMR signals of the synthesized monomer MBAA.

The ^1^H-NMR spectra of the compound are shown in Figures S1–S3. ^1^H-NMR spectra were consistent with the synthesized structure. The ^1^H-NMR spectral data of the compound were recorded in DMSO taking TMS as an internal standard. The ^1^H-NMR spectra of the MBAA exhibited expected signals at *δ*4.74, *δ*4.89, *δ*5.72–5.69 and *δ*6.29–6.22 ppm, respectively. The protons of –CH_2_ groups (–CH_2_–NH) of the MBAA gave a signal at *δ*4.74 ppm. The proton of –NH groups (–NH–CH_2_) gave a signal at 4.89 ppm. The –CH_2_ (–CH_2_=CH) and –CH (–CH=CH_2_) proton signals were appeared at *δ*5.70 and *δ*6.27 ppm, respectively. The ^13^C-NMR spectral data of the compound were recorded in methanol and is shown in Figures S4 and S5. The ^13^C-NMR spectrum of the compound exhibited the expected main signals (Scheme 2). The carbonyl group occured at 165.4 ppm. The methine signals were centered at 131.8 ppm and the methylene signals were centered at 126.4 ppm. Small signal assigned to methanol (43.8 ppm) was present. The methylol amide unit of the cross-linker occured as a strong signal at 40.1 ppm. Obtained data were consistent with the synthesized monomer.

### 3.3. GC/MS analysis

GC-MS analysis was performed to fully characterize the structure of the synthesized monomer. This technique is a useful method for high-sensitivity analysis of volatile organic compounds and it was used for qualitative analysis of synthesized organic compound. Since the MBAA is obtained in high purity, a single chromatogram has been obtained (Figure S6). The obtained single chromatogram was scanned in WinRep library. As a result of the screening, it was determined that the MBAA was obtained with 99.5% probability (Figure S7).

### 3.4. Termal analysis

TG analysis was used to examine the thermal stability of the MBAA (Figure 3). It was performed in the temperature range of 25–900 °C under N_2_ atmosphere at 1 atm with a heating rate of 10 °C min^−1^ on a Perkin Elmer Diamond TG/DTA. The compound is thermally stable up to 150 °C. The TGA curve exhibited two steps of weight losses above this temperature. The first weight loss between 160 and 230 °C is attributable to the loss of aliphatic alkene groups in formula unit. The second significant weight loss between 300 and 450 °C, corresponds to the decomposition of remaining carboxylic and amine groups. As shown in Figure 3, approximately 5% residue remained at the end of the analysis. ICP analysis was performed for the remained residue and no metal remains were observed. Since the TG analysis was carried out in an oxygen-free environment, the residue was carbonized by pyrolysis at 900 °C. In a related study, Yang et al.’s reported that the black residue, which are the carbonized organic species, could be observed at high temperature under N_2_ atmosphere [16]. For this purpose, after the TG analysis of MBAA, the obtained black residual solids (Figure 4(a)) were heated in muffle furnace at 900 °C under oxygen environment. It was observed that the carbonized monomer completely decomposed (Figure 4(b)). This result supported that the remained residue solid at 900 °C is carbonized solid of MBAA. TGA result is consistent with the structure of the synthesized compound.

**Table 1.** Catalytic activity results in different reaction conditions.

No.	Reaction conditions	Catalyst	Reaction time (h)	Yield (%)
1	Water – HCl	Cu(II) acetate	1.5	52
2	Water – NaHSO_4_	Cu(II) acetate	2	60
3	Water – NaHSO_4_	Cu(II) glyoxime	2	No conversion
4	Water – NaHSO_4_	Pd(II) glyoxime	2	No conversion
5	Water – NaHSO_4_	Fe(II) glyoxime	2	No conversion
6	Water – NaHSO_4_	Ni(II) glyoxime	2	No conversion
7	Water – NaHSO_4_	Cu(II) carboxylate	2	95

### 3.5. Catalytic activity study

One of the most important advantages of heterogeneous catalysts is the easy recovery from the reaction medium and their high thermal stabilities. In comparison, homogeneous catalysts usually have low thermal stability, and the catalyst cannot be easily recovered from the reaction medium by filtration. The MBAA was characterized by using HPLC. First of all, the retention time of the MBAA was determined by using technical reference MBAA. As shown in Figure 5, the main product and unknown by product were determined.

The conversion values in different catalysts are given in Table 1. MBAA in this study were synthesized by different methods. Some of them were previously presented as patent. Different evaluated catalysts, including Cu(II) and other metals have been carried out. In the old method, Cu(II) catalyst were used with concentrated hydrochloric acid and it was a short and less heat process with 52% yield. Using the 80–85 ^°^C heat process with NaHSO_4_, we obtained about 60% yields. Although there was a 8% increase of the product, reaction time was two hours. We did not show any catalytic converisons by using M-glyoxime catalysts (M:Cu(II)/Pd(II)/Fe(II)/Ni(II)) in the same catalytic conditions.

When using Cu(II)-carboxylate catalyst system, we obtained very high product conversion. The obtained maximum conversion value was 95% with that catalyst*.* According to HPLC analysis, any by-product was not observed except MBAA by using Cu(II) catalyst at the end of the catalytic studies (Figure 6). The selectivity was approximately 100% for this reaction.

To determine the reusability of the catalyst, we performed same catalytic procedures for several times. Five sequential reactions were performed for 0.10 g catalyst, using Water -NaHSO_4_ agents for 2 h at 85 °C. After every reuse, the catalyst was separated from the reaction mixture, filtered off, and washed with acetone (5 mL) and water (25 mL) then dried in a vacuum oven at 50 °C and reused. The conversion values did not decrease significantly after four tests (Table S1). These results showed that the catalyst has long usability in that reaction (Figure 7).

As shown in Table 1, no conversions were observed in catalysts containing glyoxime and different metals. The acetate groups present in the catalysts 1 and 2 are also carboxylate groups. They have shown 52 and 60% conversion values, respectively. Similarly, the catalyst formed in copper and carboxylate at number 7 showed maximum conversion value 95%. This high conversion is therefore not only due to the Cu (II) metal and carboxylate groups. It is known that Cu(II) carboxylate has a regular structure. So this high conversion is thought to originate from the morphological structure and surface area of the catalyst. As emphasized in the text, no information has been given about the catalyst structure due to make a patent application.

## 4. Conclusions

MBAA has been synthesized using acrylamide, formaldehyde and by varying the metal catalysts. The synthesized MBAA was fully characterized by the FT-IR, ^1^H NMR, ^13^C-NMR, GC/MS, TGA, and HPLC measurements. It was found that remarkable increase in the percentage of yield of MBAA, when Cu(II) carboxylate is used as a catalyst. It is important to note that this yield is highest value by using copper catalyst. The catalyst has several reusability since it is heterogeneous. This feature makes the catalyst more important than homogeneous ones and it has great industrial importance. After the patent application is completed, industrial production will be implemented on the synthesis of MBAA.

## Funding

This work was supported by Small and Medium Sized Industry and Development Organizations (KOSGEB) [project number 46-(2014/ARGE23)].

## Supplemental data

The supplemental data for this article can be obtained online at https://doi.org/10.1080/15685551.2017.1332138.

## Disclosure statement

No potential conflict of interest was reported by the authors.

## Supplementary Material

TDMP_1332138_Supplementary_Material.doc
