# Supplementary material for: A novel catalyst system for the synthesis of N,N′-Methylenebisacrylamide from acrylamide
Source: Des Monomers Polym. 2017 May 26;20(1):434–40. doi: 10.1080/15685551.2017.1332138 (PMC5798195; doi:10.1080/15685551.2017.1332138)
Supplement: TDMP_1332138_Supplementary_Material.doc [file TDMP_A_1332138_SM8804.doc]

**SUPPORTING INFORMATIONS**

[A novel catalyst system for the synthesis](http://www.google.com.tr/url?sa=t&rct=j&q=&esrc=s&frm=1&source=web&cd=6&cad=rja&uact=8&ved=0CDoQFjAFahUKEwjkoNqR4fbIAhXLWywKHbt9AGg&url=http%3A%2F%2Fotc.georgetown.edu%2Findustry%2Fcatalyst-system-for-chiral-alcohols&usg=AFQjCNHZhmOwevwIlVG-Ifs31VzniHTkVA&bvm=bv.106379543,d.bGg) of N,N'-Methylenebisacrylamide from acrylamide

Abdullah Avşar, Yener Gökbulut, Burak Ay*, Selahattin Serin

*Çukurova University, Department of Chemistry, Arts and Science Faculty, 01330, Adana, Turkey*

*Phone: +90 322 338 60 81, Fax: +90 322 338 60 70*

[*bay@cu.edu.tr*](mailto:bay@cu.edu.tr)

**Figure S1.** ^1^H-NMR spectra of MBAA (All data).

**Figure S2.** ^1^H-NMR spectra of MBAA (Detailed).

**Figure S3.** ^1^H-NMR spectra of MBAA (Detailed).

**Figure S4.** ^13^C-NMR spectra of MBAA (All data).

**Figure S5.** ^13^C-NMR spectra of MBAA (Detailed).

**(a)**

**(b)**

**Figure S6.** The GC/MS chromatogram **(a)** and spectrum **(b)** of MBAA.


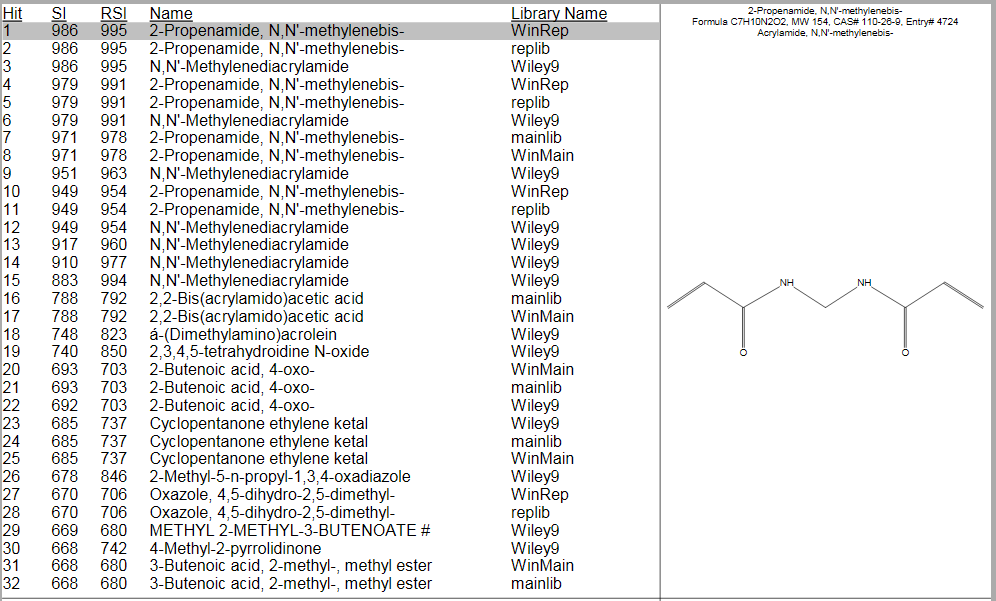


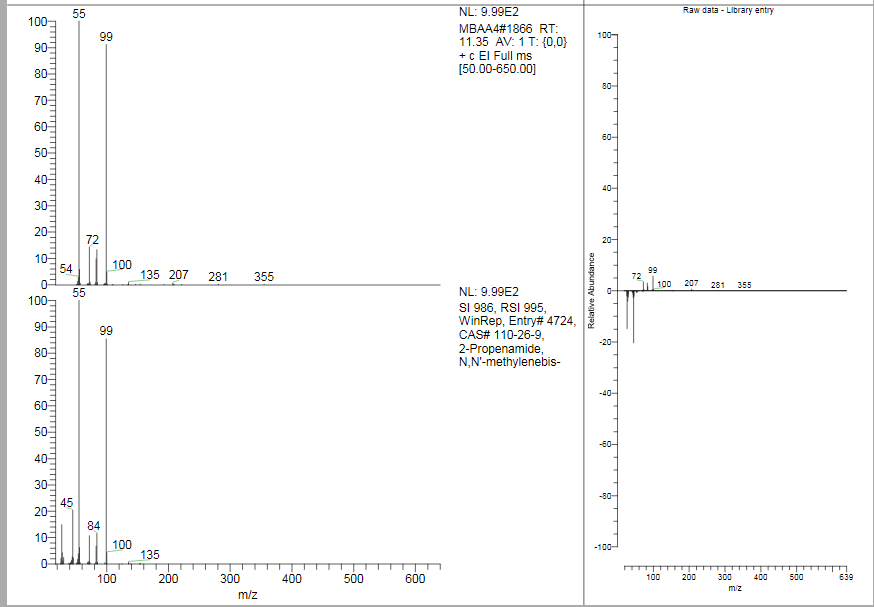


**Figure S7.** The MS spectrum analysis of the MBAA by using WinRep library.

**Table S1**

The reusability of the catalyst.

______________________________________________

| **Number of Recycle** | **Conversion (%)** |
| --- | --- |
| ____________________________________________ | |
| First reuse | 95 |
| Second reuse | 94.80 |
| Third reuse | 94.79 |
| Fourth reuse | 93.46 |
| Fifth reuse | 80.51 |
| ____________________________________________ | |
